# Involvement of Kallikrein-Related Peptidases in Normal and Pathologic Processes

**DOI:** 10.1155/2015/946572

**Published:** 2015-12-09

**Authors:** Ana Carolina B. Stefanini, Bianca Rodrigues da Cunha, Tiago Henrique, Eloiza H. Tajara

**Affiliations:** ^1^Department of Molecular Biology, School of Medicine of São José do Rio Preto, 15090-000 São José do Rio Preto, SP, Brazil; ^2^Department of Genetics and Evolutionary Biology, Institute of Biosciences, University of São Paulo, 05508-090 São Paulo, SP, Brazil

## Abstract

Human kallikrein-related peptidases (KLKs) are a subgroup of serine proteases that participate in proteolytic pathways and control protein levels in normal physiology as well as in several pathological conditions. Their complex network of stimulatory and inhibitory interactions may induce inflammatory and immune responses and contribute to the neoplastic phenotype through the regulation of several cellular processes, such as proliferation, survival, migration, and invasion. This family of proteases, which includes one of the most useful cancer biomarkers, kallikrein-related peptidase 3 or PSA, also has a protective effect against cancer promoting apoptosis or counteracting angiogenesis and cell proliferation. Therefore, they represent attractive therapeutic targets and may have important applications in clinical oncology. Despite being intensively studied, many gaps in our knowledge on several molecular aspects of KLK functions still exist. This review aims to summarize recent data on their involvement in different processes related to health and disease, in particular those directly or indirectly linked to the neoplastic process.

## 1. Introduction

Human kallikrein-related peptidases (KLKs) are a subgroup of serine proteases that have important roles in regulating normal physiological functions, such as immune response, skin desquamation, enamel formation, and semen liquefaction, and the corresponding pathological conditions. There is growing evidence in the literature supporting the view that KLKs are also implicated in tumorigenesis by activating proteolytic processes associated with the neoplastic phenotype. The potential mechanisms involved include the modulation of growth factor bioavailability and activation of hormone and protease-activated receptors (PARs) resulting in proliferative signaling pathways, the degradation of extracellular matrix, cleavage of junction proteins and induction of an epithelial-mesenchymal transition (EMT) phenotype leading to increased tumor cell migration and invasion, and the modulation of interactions between cancer cells and their microenvironment promoting angiogenesis and other protumorigenic processes (reviewed by [1–3]).

The potential of KLKs as cancer markers has been suggested for several members of this protease family [2, 4–6], particularly for kallikrein-related peptidase 3 or prostate-specific antigen (PSA) [7]. PSA is well accepted for assessing recurrence risk in patients with prostate cancer, but its predictive power for diagnosis has been questioned, since several factors other than malignancy may be associated with its high levels in serum, such as preanalytical variables, benign diseases, and drugs [8, 9]. Biomarker panels combining PSA and other promising markers, including members of the KLK family, are expected to improve prostate cancer screening and reduce unnecessary treatments, a strategy that may also be used for detection and monitoring of other malignancies and nonmalignant diseases.

In this paper, we review the current knowledge about the evolution and functions of human kallikrein-related peptidases, their substrates, and their role in health and disease, particularly in the context of cancer.

## 2. The Human Degradome

Protein synthesis is essential for living, metabolically active cells, but its counterpart, protein degradation, is no less important. Proteolytic mechanisms driven by proteases maintain appropriate protein levels and recognize and degrade the misfolded or mislocalized ones. In addition to acting in nonspecific catabolism, proteases are involved in selective cleavages and activations, modulating protein-protein interactions and contributing to cell signaling both as catalytic units and as multicatalytic complexes. Due to their broad-spectrum actions, proteases play critical roles in regulating normal biological processes, including DNA replication and transcription, cell proliferation, differentiation, and apoptosis. When altered, they may facilitate the development of pathological conditions such as inflammatory and degenerative disorders (reviewed by [10]). The importance of these hydrolytic enzymes is reflected by the number of genes already identified in several mammalian species, with more than 500 in human and primates and even more in rodents [11–14].

The complete set of human proteases—named the human degradome—is distributed in aspartic-, threonine-, cysteine-, serine-, and metalloprotease classes according to the chemical group involved in their catalytic activity [15, 16], and the latter three are the most populated classes [10]. Their substrate cleavage patterns may be specific for a single peptide, as in the case of proteases involved in signaling pathways, or common for a broad range of peptides, which is well exemplified by digestive enzymes [17]. Otherwise, inactive proteases or pseudoproteases bind to their cognate substrate without cleaving them, thus exerting a regulatory function [18].

Detailed information on proteases in prokaryotes and eukaryotes, protease families, pseudogenes, the sequences derived from endogenous retroviruses, 3D structures, substrates, and proteolytic events has been accumulated in different databases such as MEROPS [19] and Degradome [20].

## 3. The Serine Protease Group

Approximately one-third of proteolytic enzymes are serine proteases, usually endopeptidases. These enzymes use the serine residue present in their active site as a nucleophile to attack the peptide bond of the substrate [21]. In humans, many serine proteases are involved in extra- and intracellular processes mainly related to food digestion, blood coagulation, and immunity (reviewed by [1, 22]). Although these processes are essential for the purposes of catabolism or selective cleavages required for cell signaling, serine protease activity (as well as that of other proteases) is potentially devastating, and several cellular mechanisms were selected to modulate and keep them within limits. For example, they are stored as inactive zymogens or inside granules and can access the substrates only through controlled actions. In addition, serpins, a superfamily of serine protease inhibitors, antagonize their activities in many metabolic pathways, arresting the proteases into an irreversible complex (reviewed by [1]).

Although tightly controlled, several serine proteases have been associated with human diseases. For example, high granzyme levels (granule-secreted enzymes found in cytotoxic T cells and natural killer cells) have been observed in chronic inflammatory diseases such as rheumatoid arthritis [23], asthma [24], diabetes [25], atherosclerosis [26], and chronic obstructive pulmonary [27] and cardiovascular diseases [28]. They have also been implicated in susceptibility to skin tearing and disorganized collagen as observed in chronic wounds and aged/sun-damaged skin (reviewed by [29]). The role of granzymes in these conditions resides in their ability to cleave many substrates, inducing apoptosis through caspase-dependent and caspase-independent pathways [30]. Their potential to create or destroy autoimmune epitopes [31] and be improperly regulated in chronic wounds or released nonspecifically from immune cell into extracellular spaces also contributes to chronic inflammation or extracellular matrix disorganization [27, 32].

Increased levels of neutrophil proteases such as elastase, cathepsin G, and myeloblastin have also been correlated with the severity of cystic fibrosis and chronic obstructive pulmonary disease [33]. Similarly, tryptase and chymase, two serine proteases stored in mast cell granules, take part in the pathophysiology of asthma [34], psoriasis [35], atherothrombosis [36], and fibrotic [37] and inflammatory kidney diseases [38].

With respect to cancer, several serine proteases have been linked to tumor development and progression by activating proteolytic processes that are associated with the neoplastic phenotype (reviewed by [1]). Specifically, a family of serine proteases expressed and secreted in many tissues participates in complex networks of cell signaling pathways that are related to cancer [4–7]. One of the most useful cancer biomarkers in clinical medicine is kallikrein-related peptidase 3 or PSA, which is a member of this family (reviewed by [7]), and there is evidence that other KLKs are also deregulated in cancer and other diseases [4, 39–147] as summarized in Table 1.

## 4. The Human Kallikreins

Human kallikreins, initially detected at high levels in pancreas,* kallikreas* in Greek, include plasma and tissue serine proteases, which are two categories that differ in molecular weight, substrate specificity, and gene structure. The unique plasma kallikrein (PKK) is a glycoprotein encoded by the* KLKB1* gene on chromosome region 4q35 and is predominantly synthesized in the liver as an inactive precursor. After activation by the coagulation factor XII, PKK cleaves high molecular weight kininogen to release bradykinin, a mediator of blood coagulation, inflammation, blood pressure, and thrombosis risk [148].

### 4.1. Kallikrein-Related Peptidases at DNA Level: Genomic Organization and Evolutionary Aspects

**Figure 1:**
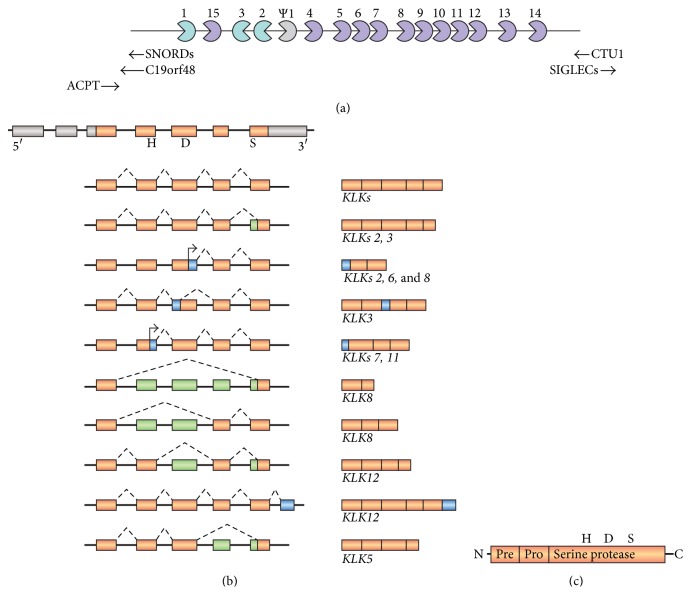
*KLK* gene cluster and schematic representation of the human* KLK* gene and protein structure. (a)* KLK* gene cluster on the 19q13.33–13.41 chromosome region including the pseudogene* KLKP1* and the transcriptional direction from centromere to telomere, except for* KLK2* and* KLK3*, which have the opposite transcriptional direction. The classic* KLK* genes (*KLKs 1–3*) are turquoise,* KLK4–KLK15* are medium purple, and the* Ψ KLK1* processed pseudogene is silver; the arrowheads represent the neighboring genes:* ACPT* (testicular acid phosphatase) and the Siglec (sialic acid-binding immunoglobulin-like lectin) gene family as well as other less characterized genes (*SNORDs*,* C19orf48*, and* CTU1*). (b) The human* KLK* gene consists of 5 coding exons (orange boxes represent coding exons; silver boxes represent noncoding exons) and their 4 intervening introns. The positions of the catalytic residues are highly conserved with the histidine (H), aspartic acid (D) 3, and serine (S) codons on coding exons 2, 3, and 5, respectively. Most* KLK* genes demonstrate alternative splicing, which generates several transcript variants. Alternative 3′ splice sites or skipped exons (shown in green) result in short variants of* KLKs 2*,* 3*,* 5*,* 8*, and* 12 genes*. Alternative 5′ splice sites or start sites (shown in blue) also generate short variants of* KLKs 2*,* 3*,* 6*,* 7*,* 8*, and* 11* genes. Utilization of the alternative exon 6 generates a long transcript encoding a variant of* KLK12* gene (shown in blue). (c) KLK proteins are single-chain proteases that are synthesized as preproenzymes and are proteolytically processed to pro-KLKs and secreted after removal of the terminal signal peptide (Pre). The KLK sequence also includes a propeptide (Pro) that maintains the inactive state of the enzyme, as well as a serine protease domain.

The 15 tissue kallikreins or kallikrein-related peptidases (KLKs) are encoded by genes that are tightly clustered in an approximately 300 kb sequence of the 19q13.33–13.41 chromosome region, all containing 5 coding exons with comparable lengths and sequence homology [149, 150]. A pseudogene (*KLKP1*) has also been assigned to this region [151], as well as multiple repetitive elements such as ALU, Tigger2, MER8, and MSR1 [152]. The large contiguous human* KLK* gene cluster is limited by the* ACPT* (testicular acid phosphatase) gene and the Siglec (sialic acid-binding immunoglobulin-like lectin) family of genes at centromeric and telomeric positions, respectively, and other less characterized genes (*SNORD88C*,* C19orf48*,* MGC45922*, and* CTU1*) (Figure 1).

**Figure 2:**
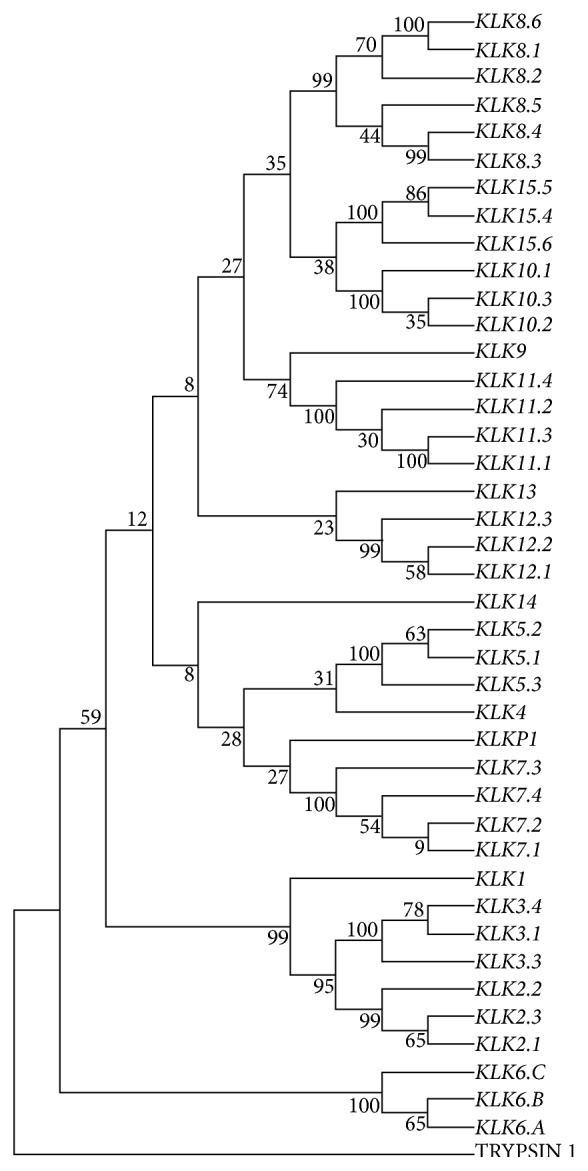
Phylogenetic relationships within the human tissue* KLK* gene family in humans. Phylogenetic analysis was performed using the MEGA5 [205] and Maximum Likelihood methods based on the GTR model (*General Time Reversible*) [154] with Gamma distribution. The bootstrap method was used (with 1000 data set replicates) to investigate node robustness [206]. The phylogenetic tree includes 15 KLK transcripts, the pseudogene-1 (*KLKP1*) sequence, and the trypsin 1 gene sequence (*PRSS1*) [155, 156]. The sequences were obtained from the NCBI Reference Sequence (RefSeq) database (http://www.ncbi.nlm.nih.gov/). Numbers indicate the percentage of 1000 bootstrap replicates at each node in the consensus. Bootstrap value ≤95.

The colocation and sequence conservation in a wide variety of species make this human tissue serine proteinase family a very interesting target for evolutionary studies [153]. The phylogenetic analysis of* KLK*s performed by the Maximum Likelihood method [154], using the transcript isoforms of 15* KLK* genes, the pseudogene-1 (*KLKP1*) sequence, and the* PRSS1* (trypsin 1) transcript sequence as an external group, reveals five major branches: (a) the classic* KLKs* (*KLKs 1*–*3*), (b)* KLKs 4*,* 5*,* 7*, and* 14 *and* KLKP1*, (c)* KLKs 9 *and* 11*, (d)* KLKs 8*,* 10*, and* 15*, and (e)* KLKs 12 *and* 13*, and a separate branch with* KLK6*. The tree (Figure 2) is similar in several aspects to other phylogenetic analyses of this cluster [150, 153, 155–157] but also includes the isoforms and reinforces the idea that all* KLK* genes evolved from a single gene by successive tandem duplications and genomic rearrangements facilitated by repetitive elements.

The high similarity between* KLK2* and* KLK3* sequences and the highest support value also suggest that they might have formed by duplication later in evolution. The data grouping* KLK4/KLK5* and* KLK9/KLK11* also corroborate previous studies [153, 156]. The isolated position of* KLK6* in this phylogenetic tree, unlike the findings of other authors, may explain the apparent distance of the remaining family members in respect to normal and pathological functions.

### 4.2. Kallikrein-Related Peptidases at RNA Level: Transcriptional Regulation Mechanisms

Kallikrein-related peptidase expression is regulated at transcriptional, translational, and posttranslational levels. At the transcriptional level, several response elements (REs) have been identified in the* KLK* promoters such as an estrogen-related receptor *γ* (ERR*γ*) response element [158], a GATA binding motif in* KLK1* [159], and functional retinoic acid response elements (RAREs) in* KLK10* [160]. Due to the importance of* KLK3* expression in prostate cancer, a number of REs have already been described for its promoter, including Sp1/Sp3 [161] and WT1 transcription factor-binding sites [162], a putative p53 RE [163], an XBE (X-factor-binding element that binds specifically to the NF-kappaB p65 subunit) in the AREc (androgen response element enhancer core) [164], and androgen-responsive elements (AREs), the last of which were also present in the* KLK2* promoter (reviewed by [127, 165]).

*KLK* gene expression can also be regulated by epigenetic mechanisms, including histone modifications such as DNA methylation as well as microRNAs (reviewed by [166]), which can affect normal cell physiology and facilitate tumorigenesis if altered. In fact, aberrant promoter methylation leading to* KLK10* downregulation has been described in acute lymphoblastic leukemia [167] as well as in breast [168], gastric [91], and prostate cancer [169]. Similarly, abnormal histone acetylation at* KLK2* and* KLK3* sequences and deregulated expression of miRNAs targeting* KLK *genes have also been reported in kidney, prostate, and breast cancer cell lines (reviewed by [166]).

In addition to epigenetic events, polymorphisms in regulatory sequences can potentially alter RNA transcription rates and protein levels, as was observed for the homozygous G base substitution (rs266882) in the androgen response element (ARE-1) of the* KLK3* promoter [170] and for polymorphic alleles in the 5′-flanking region of the* KLK1* gene [171].* KLK* gene activity is likewise affected by polymorphisms in the coding region or in the 3′-UTR and downstream sequences of the* KLK1*,* KLK2*,* KLK3*, and* KLK7* genes (reviewed by [47]).

According to the NCBI Reference Sequence Database (accessed in November 20, 2014), with the exceptions of* KLK1*,* KLK4*,* KLK9*,* KLK13*, and* KLK14*, human* KLK* genes have multiple isoforms. The alternative transcripts apparently are species specific [155], and a number of them are cancer specific (reviewed by [172]), which supports the idea that they are constantly evolving. The diversity of these isoforms, especially those with no peptidase catalytic motifs, may indicate a type of activity control, for example, by competing for the same substrates or performing different tissue-specific functions [155].

### 4.3. Kallikrein-Related Peptidases at Protein Level

**Figure 3:**
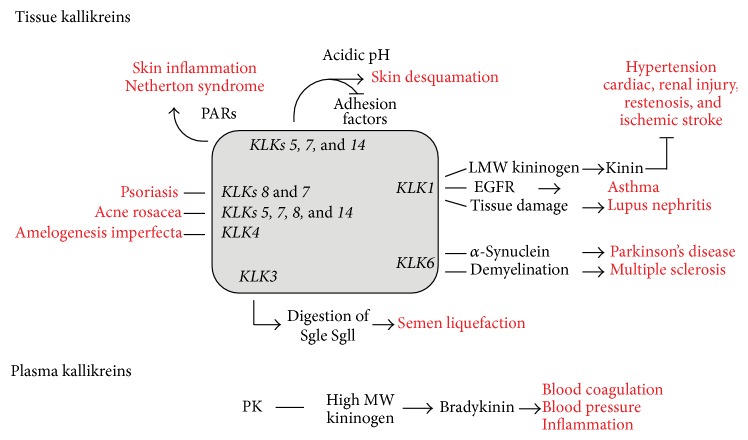
Schematic representation of KLK functions related to physiological and pathological conditions. KLKs are involved in several normal processes including blood pressure, coagulation, semen liquefaction, and skin desquamation and can also protect against cardiac injury and ischemia. These proteases may also participate in skin inflammation, neurodegeneration, and autoimmune diseases.

The KLKs are proteins of 230 amino acids and 28 to 33 kDa, although some small isoforms reach only 3 kDa. Their standard tertiary structure consists of two juxtaposed six-stranded antiparallel *β*-barrels and two *α*-helices with the active site between the barrels [173, 174]. They are synthesized as preproenzymes, which are proteolytically processed to pro-KLKs and secreted after removal of the terminal signal peptide. Their ability to release kinins was initially viewed as the definition of a true kallikrein. However, besides plasma kallikrein, only KLK1 has the ability to cleave kininogen (in this case, low molecular weight kininogen) to release kinin. The tissue kallikrein-kinin system can protect against cardiac injury and ischemia/reperfusion-induced cardiomyocyte apoptosis as well as against oxidative stress-induced renal cell apoptosis via stimulation of kinin B2 receptor-Akt [175]. Otherwise, this system appears to be involved in the development of lupus nephritis by increasing local tissue damage triggered by autoimmune inflammation [176] (Figure 3).

As mentioned above,* KLK* promoters have several hormone response elements, and their expression can be regulated by steroid hormones [177]. Therefore, KLK levels in different tissues are dependent not only on the presence of specific transcriptional and translational regulators, but also on proteolytic mechanisms, as previously referred to in the degradome section. Shaw and Diamandis [178] detected distinct expression profiles for several kallikrein-related peptidases: KLK1 was highly expressed in the pancreas and salivary gland, KLKs 2, 3 (also observed in seminal plasma), and 11 were highly expressed in the prostate, KLK5 was expressed in the skin, KLK6 was expressed in the brain, KLK9 was expressed in the heart, and KLK12 was expressed in several anatomical sites. KLKs 4, 8, 14, and 15 exhibited a more homogeneous profile or were not detected in various tissues. Komatsu et al. [179] analyzed the skin stratum corneum and identified the presence of many KLKs (KLKs 5–8, 10, 11, 13, and 14). Generally, expression patterns are compatible with their origins—duplicate genes have similar expression patterns in the same tissues, and coexpression patterns are compatible with their physiological functions [153].

## 5. Kallikrein-Related Peptidases and Their Relationship to Health and Disease

### 5.1. Normal Physiological Processes and Nonmalignant Diseases

Similar to what has been observed for other proteases, several regulatory mechanisms protect tissues from harmful proteolysis by KLKs. In addition to controlled proenzyme activation and endogenous inhibitors (such as *α*_2_-macroglobulin and serpins), there are also inactivating cleavages and allosteric regulation (reviewed by [165]). Regulatory steps may be performed by other proteases including members of the KLK family, which are supported by their coexpression in the same tissue. For example, a KLK cascade including KLK2, KLK14, and probably other KLKs activates pro-KLK3 to generate the mature proteinase that directly cleaves the semenogelins SgI and SgII resulting in seminal clot liquefaction and spermatozoa release [180]. Recently, Yoon et al. [181] observed that MMP-20, which is usually expressed only in dental enamel, processes the prosequence of nine different* KLKs* and may be a nonspecific activator of the* KLK* family in pathological conditions.

Another proteolytic cascade has been described for the skin desquamation process in which KLK5 may be autoactivated or activated by KLK14 at neutral pH and then process KLK7, regulating skin desquamation. This cascade may start by KLK6 autoactivation following the cleavage of KLK11, which in turn activates KLK14. Although not completely understood, skin desquamation also depends on other proteases, including cathepsins, aspartic proteases, urokinase, plasmin, and the inflammatory metalloproteinases. Because KLK regulation is critical for proper desquamation, various endogenous inhibitors participate as attenuators of their activities, mainly LEKTI (serine protease inhibitor Kazal-type 5), a protein encoded by the* SPINK5* gene. Other factors such as an acidic environment and UV irradiation (and resulting inflammation) may inhibit LEKTI, also contributing to increased KLK expression and enhanced desquamation [64]. The lack of LEKTI expression in Netherton syndrome, a rare genetic skin disease characterized by congenital ichthyosis and severe allergic manifestations, indeed results in increased proteolytic activities of KLK5 and KLK7, which trigger an inflammatory process by activating protease-activated receptor-2 (PAR-2) and stimulating cytokine production [70] (Figure 3).

KLK deregulation is also observed in several other pathological conditions, of which neurodegenerative disorders are good examples (Figure 3). Alzheimer's disease (AD) and Parkinson's disease (PD) are the most prevalent human neurodegenerative disorders. Both are caused by the aggregation of proteins: AD is characterized by extracellular deposits of amyloid *β* (A*β*) and intraneuronal aggregates of tau protein in specific brain regions, and PD is characterized by intracellular neuronal deposits (Lewy bodies and neurites) formed by insoluble *α*-synuclein [182, 183].

There is convincing evidence from the literature on Alzheimer's disease that KLK6, the most abundant kallikrein-related peptidase in the central nervous system, cleaves the amyloid precursor protein (APP), a transmembrane glycoprotein from which A*β* derives. The proteolytic activity of KLK6 against APP and substrates in the extracellular matrix and perineuronal net places this peptidase as a potential component of AD pathogenesis. KLK6 expression is reduced in brain tissues, as well as in cerebrospinal fluid of AD patients [42, 184, 185], but the mechanisms behind these findings and their functional consequences are not yet known. Actually, other enzymes (*α*-, *β*-, and *γ*-secretases) cleave APP in different sites and generate several fragments; some of them are aggregation-prone [183]. KLKs may, for example, promote a bias toward synthesis of these toxic fragments by *β*- and *γ*-secretases.

Besides KLK6, the kallikrein-related peptidases 7 and 10 show decreased and increased levels, respectively, in cerebrospinal fluid of AD patients [39]. Recently, Shropshire and collaborators observed that KLK7 is able to cleave the core of A*β* in vitro, inhibiting A*β* aggregation and reducing neuronal toxicity [186]. This result may open new opportunities towards treatments for AD.

Several studies on Parkinson's disease have implicated KLK6 in the degradation of intracellular *α*-synuclein [187]. Recent data suggested that secreted *α*-synuclein is also involved in the development of PD by affecting neuronal cell viability [188] and activating inflammatory response [189]. Although still controversial with respect to the intracellular type, KLK6 inefficiency in *α*-synuclein degradation seems to contribute to PD pathogenesis, probably due to an altered trafficking of KLK6 [187, 190] or to the resistance of certain forms of *α*-synuclein to KLK6-proteolysis [76, 191].

Multiple sclerosis (MS) is another example of neurodegenerative disorder in which KLK6 levels are altered. In MS patients, KLK6 is abundantly expressed and cleaves myelin proteins, resulting in demyelination and oligodendrogliopathy [192].

As may be noted from AD, PD, and MS data, KLK6 seems to be important for the neuronal homeostasis and survival. However, other kallikrein-related peptidases are probably involved in these processes, as can be deduced from the data on overexpression of KLK1 in epilepsy [193] and on the ability of a set of KLKs (KLK1, KLKs 5–7, and KLK9) to promote neural injury [62].

### 5.2. Malignant Diseases

**Figure 4:**
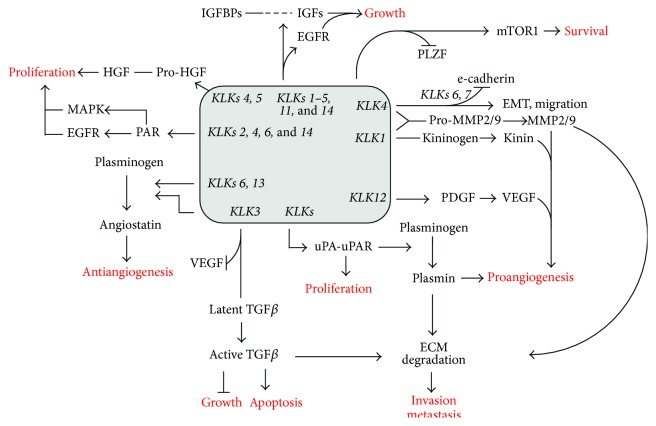
Kallikrein-related peptidases and cancer. KLKs participate in proteolytic pathways that contribute to the neoplastic process by facilitating cell proliferation via growth factors and modulating cell survival through mTOR signaling. They can also regulate angiogenesis, cell migration and invasion by angiogenic vascular endothelial growth factor (VEGF) secretion, metalloproteinase activation, extracellular matrix (ECM) degradation, and epithelial-mesenchymal transition (EMT) induction. However, KLKs also have a protective effect against cancer, promoting apoptosis or inhibiting angiogenesis and cell proliferation.

As evidenced by the literature, particularly in prostate cancer, KLKs participate in proteolytic pathways that contribute to the neoplastic process (Figure 4). With respect to tumor growth, KLK1 facilitates EGFR and ERK1/2 cascade activation, which is involved in cell proliferation [194]. Similarly, KLK1, KLK2, and KLK3 can regulate tumor growth through IGF-binding protein (IGFBP) degradation, thereby allowing the release of the insulin-like growth factors (IGFs) and proliferative signals. However, a negative regulatory role for KLK3 in cancer has also been suggested because this protease can activate latent transforming growth factor-*β* (TGF*β*), a known suppressor of growth and promoter of apoptosis [2].

Recent data have demonstrated that kallikrein-related peptidase 4 and its substrate, promyelocytic leukemia zinc finger protein (PLZF), modulate androgen receptor (AR) and mTOR signaling in prostate cells to regulate cell survival. In fact, KLK4 negatively regulates PLZF, thus preventing its binding and inhibition by AR, which keeps mTORC1 signaling active and ensures cell survival [195].

During neoplastic progression, different KLKs can regulate new vessel formation, which are essential to provide oxygen and nutrients to proliferating cancerous cells. KLKs 1 and 4 stimulate angiogenesis by cleaving kininogen to kinin or activating prometalloproteinases 2 and 9 to their active forms, thereby potentiating extracellular matrix hydrolysis and enabling endothelial cell migration and neovascularization [196–198]. Other kallikrein-related peptidases (KLKs 2 and 4) can stimulate the urokinase plasminogen activator (uPA)/uPA receptor system, which also leads to metalloproteinase activation and extracellular matrix degradation [2]. KLK12 may then promote angiogenesis by the conversion of the membrane-bound platelet-derived growth factor B (PDGF-B) precursor into a soluble form that modulates secretion of the angiogenic vascular endothelial growth factor A (VEGF-A) [199]. Some KLKs, such as KLKs 3, 6, and 13, have the opposite action by blocking VEGF and/or fibroblast growth factor 2 (FGF2) or generating angiostatin-like fragments from plasminogen, which are potent inhibitors of angiogenesis in vitro [2].

Tumors have an increased acidic microenvironment resulting from accelerated glycolysis and lactate accumulation and thus low pH in the extracellular space [200]. Because an acidic environment may block the kallikrein inhibitor LEKTI, contributing to increased KLK expression and loss of cellular adhesion in skin desquamation, it is reasonable to consider a similar mechanism during neoplastic dissemination [201]. In fact, the metastatic process is associated with a transition from tightly connected cells to cells with increased motility, namely, the epithelial-mesenchymal transition, where KLKs play important roles. For example, KLKs activate latent TGF*β*, which induces EMT, and are associated with the loss of E-cadherin in tumor cells and thus with decreased cell-cell adhesion [202]. They also trigger extracellular matrix degradation via prometalloprotease activation and hence promote tissue invasion [2].

These examples demonstrate how important kallikrein-related peptidases are in tumor development and progression. The biological processes in which they participate are related to diseases other than cancer but are directly connected with cancer pathways, including cell proliferation, adhesion, inflammation, and apoptosis.

## 6. Therapeutic Relevance of KLKs

As discussed in previous sections, KLKs have been associated with different pathologic processes, from skin diseases to neurodegenerative disorders and cancer. The progress in our knowledge on all members of this protein family, functions, 3D structures, substrates, and physiological roles, has provided opportunities to develop new therapeutic approaches for different disorders.

KLKs are targeted by several types of inhibitors, including small-molecule inhibitors, antibody-, protein-, and peptide-based inhibitors, KLK-activated prodrugs, interfering RNAs, and immunotherapeutic vaccines (reviewed by [3]). PROSTVAC, for example, is a prostate cancer vaccine consisting of a KLK3 recombinant vector that contains transgenes for three T-cell costimulatory molecules (TRICOM). This vaccine has demonstrated success in inhibiting, with few side effects, cell proliferation and tumor growth and in improving overall survival [203].

Prodrugs activated by KLKs are another strategy that has been investigated. For instance, KLK3-activated peptides have the ability to target the prostate since most KLK3 is expressed in the gland whereas circulating KLK3 is normally inactivated in plasma by endogenous inhibitors [204]. This drug has overcome the challenge of specificity, although similar successful results are not always achieved. The reasons for that include the fact that the active sites of members of KLK family are conserved, which hampers drug design. The resolution of 3D structure of KLKs should help in this regard. However, KLKs also have overlapping and even opposing actions, which certainly depend on the physiologic, tissue, and disease context [203].

## 7. Conclusions

The KLK network is impressive. Its intricate signaling pathways and protein interactions strongly show that this group of proteases contributes to normal and pathological metabolisms. However, despite being intensively studied, there are many gaps in our knowledge on the molecular aspects of the KLK family. For example, there is no doubt that KLK expression deregulation participates in the development of neurodegenerative disorders. But what exactly is its role? Would it be a primary and direct one, promoting erroneously protein degradation, which results in pathogenic fragments? Or would it be one that implies cooperating with specific secretases and other enzymes to generate toxic deposits?

In cancer, it is not clear whether KLKs alterations are driver mutations or deleterious passenger mutations. The fact that similar sets of KLKs are associated with different tumor types and facilitate proliferation, migration, and other cancer hallmarks aligns with driver mutations. Differently, antiproliferative effects of KLKs and similar regulatory factors for different members of this family may argue in favor of random passenger mutations. However, both statements are not mutually exclusive and may occur simultaneously or sequentially. In fact, the idea of sequential occurrence is interesting: considering the complexity of human proteolytic system, it is reasonable to assume that the expression of specific KLKs may counteract the under- or overexpression of other KLKs or enzymes or even that those KLKs are activated, one after the other, to neutralize the expression of a driver mutation, but without success. The analysis of KLK panels in large sets of samples from diverse stages of the disease, including premalignant phases, will probably help to reveal how the expression profile evolves during the course of the disease.

Many questions are still unanswered and the scenario is therefore incomplete. Many more data are necessary to improve our understanding on the function, substrates, and role of KLKs in health and disease in order to distinguish in each case whether they are heroes, villains, or supporting actors.

## Figures and Tables

**Table 1:** Kallikrein-related peptidases. Gene expression pattern, SNPs, and promoter methylation related to cancer and other diseases. CSF = cerebrospinal fluid.

Disease	Kallikrein	Factor	Observation	Reference
Alzheimer's disease	*KLKs 6 (CSF), 10*	Increased expression		[39–42]
	*KLKs 6 (brain, blood), 7*	Decreased expression		

Amelogenesis imperfecta	*KLK4*	Mutation	Disease-causing mutation	[43–45]

Aneurism	*KLK6*	Decreased expression	Suggestion of unfavorable prognosis	[46, 47]
	*KLK8*	SNP	Suggestion of unfavorable prognosis	

Asthma	*KLK3*	SNP		[48]

Atopic dermatitis	*KLK5*	Decreased expression		[49, 50]

Bipolar disease	*KLK8*	SNP	Suggestion of unfavorable prognosis	[51]

Coronary artery disease	*KLK1*	SNP	Controversial prognosis	[52, 53]
	*KLK1*	Increased expression	Predictor of disease	

Kidney disease				
Lupus nephritis	*KLK1*	SNP	Disease-associated SNP	
Acute kidney injury	*KLK1*	SNP	Suggestion of unfavorable prognosis	[54–56]
Diabetic nephropathy	*KLK1*	Increased expression	Tubular inflammation	

Multiple sclerosis	*KLK6*	Increased expression	Advanced disease	[57–59]

Dementia with Lewy bodies	*KLK6*	Decreased expression	Suggestion of diagnostic marker	[60]

Other neurodegenerative diseases	*KLKs 1*,* 5*,* 6*,* 7*, and* 9*	Increased expression	Suggestion of disease-associated marker	[61–63]

Other skin diseases	*KLKs 5–8*,* 10–13*, and* 15*	Increased expression	Suggestion of unfavorable prognosis	[64–73]
Netherton syndrome	*KLK5*	Increased expression	Suggestion of unfavorable prognosis	

Psoriasis	*KLK8*	Increased expression	Suggestion of unfavorable prognosis	[74, 75]
	*KLKs 6*,* 8*,* 10*, and* 13*	Increased expression	Severity of skin lesions	

Parkinson's disease	*KLK6*	Increased expression	Disease-associated marker	[76]

Sjogren disease	*KLK11*	Increased expression	Suggestion of disease-associated marker	[77]

Breast cancer	*KLKs 2*,* 4*	SNP	Breast cancer risk	
	*KLK3*	SNP	Association with less aggressiveness	
	*KLKs 5*,* 10*, and* 14*	Increased expression	Potential diagnostic biomarkers	
	*KLKs 6*,* 12 variant 3*, and* 15*	Increased expression	Suggestion of favorable prognosis	[4, 47, 78–83]
	*KLKs 3*,* 8*, and* 12*	Decreased expression	Suggestion of favorable prognosis	
	*KLKs 5*,* 7*	Increased expression	Suggestion of unfavorable prognosis	
	*KLK10*	Methylation	Suggestion of favorable prognosis	

Cervix cancer	*KLK7*	Increased expression	Controversial prognosis	[84, 85]

Colorectal cancer	*KLKs 4*,* 6*,* 7*, and* 10*	Increased expression	Suggestion of unfavorable prognosis	[86–90]

Gastric cancer	*KLKs 6*,* 7*, and* 10*	Increased expression	Suggestion of unfavorable prognosis	
	*KLK13*	Increased expression	Suggestion of favorable prognosis	[90–95]
	*KLK11*	Decreased expression	Suggestion of unfavorable prognosis	

Head and neck cancer	*KLK10*	Methylation	Suggestion of unfavorable prognosis	[96–99]
	*KLKs 4–8*,* 10*	Increased expression	Suggestion of unfavorable prognosis	

Intracranial tumor	*KLKs 6–8*	Increased expression	Controversial prognosis	[100, 101]

Lung cancer	*KLK10*	Methylation		[102–106]
	*KLKs 5–7*	Increased expression	Suggestion of unfavorable prognosis	
	*KLKs 11*,* 13*, and* 14*	Increased expression	Diagnostic marker	
	*KLKs 8*,* 12*	Decreased expression	Suggestion of unfavorable prognosis	

Melanoma	*KLKs 6*,* 8*, and* 13*	Increased expression		[107, 108]
	*KLK7*	Increased expression	Suggestion of favorable prognosis	

Ovarian cancer	*KLKs 4*,* 6*	Increased expression	Advanced stage	
	*KLKs 8–10*,* 11*,* 13*, and *14*	Increased expression	Suggestion of favorable prognosis	
	*KLKs 5*,* 7*	Increased expression	Suggestion of unfavorable prognosis	[47, 78, 109–125]
	*KLK10, KLKP1*	SNP		
	*KLKs 3*,* 15*	SNP	Suggestion of unfavorable prognosis	

Pancreatic cancer	*KLK7*	Increased expression	Controversial prognosis	[126]

Prostate cancer	*KLK3*	Increased expression	Disease monitoring and recurrent prediction	[47, 78, 127–147]
	*KLKs 1*,* 2*,* 4*, and* 15*	Increased expression		
	*KLKP1*	Decreased expression		
	*KLK7*	Increased expression	Controversial prognosis	
	*KLK11*	Decreased expression	Suggestion of unfavorable prognosis	
	*KLKs 2*,* 3*,* 4*, and* 10*	SNP	Suggestion of unfavorable prognosis	
	*KLK12*	SNP	Cancer predisposition	
	*KLKs 4*,* 14*, and* 15*	SNP	Suggestion of unfavorable prognosis	
